# Elective pelvic irradiation in prostate cancer patients with biochemical failure following radical prostatectomy: A propensity score matching analysis

**DOI:** 10.1371/journal.pone.0215057

**Published:** 2019-04-11

**Authors:** Changhoon Song, Sang Jun Byun, Young Seok Kim, Hanjong Ahn, Seok-Soo Byun, Choung-Soo Kim, Sang Eun Lee, Jae-Sung Kim

**Affiliations:** 1 Department of Radiation Oncology, Seoul National University College of Medicine, Seoul National University Bundang Hospital, Seongnam, Republic of Korea; 2 Department of Radiation Oncology, Dongsan Medical Center, Keimyung University School of Medicine, Daegu, Republic of Korea; 3 Department of Radiation Oncology, Asan Medical Center, University of Ulsan College of Medicine, Seoul, Republic of Korea; 4 Department of Urology, Asan Medical Center, University of Ulsan College of Medicine, Seoul, Republic of Korea; 5 Department of Urology, Seoul National University College of Medicine, Seoul National University Bundang Hospital, Seongnam, Republic of Korea; University of Toronto, CANADA

## Abstract

**Purpose:**

To investigate whether whole pelvic radiotherapy (WPRT) improves biochemical relapse-free survival (bRFS) vs. prostate bed radiotherapy (PBRT) in prostate cancer patients receiving salvage radiotherapy (SRT) after radical prostatectomy.

**Methods:**

Data from patients with prostate cancer who underwent SRT for biochemical recurrence between 2005 and 2012 in two academic institutions were retrospectively reviewed. Patients treated with WPRT in one hospital were compared with patients treated with PBRT in the other. Propensity scoring was performed to balance the characteristics of the different treatment groups, and bRFS was compared.

**Results:**

Data from a total of 191 patients were included in the analysis (WPRT, n = 108; PBRT, n = 83). The median follow-up period was 66 months. Prior to matching, patients who received WPRT had higher pathologic Gleason scores as well as a higher incidence of pre-SRT PSA levels >0.5 ng/mL and lower rates of concurrent androgen-deprivation therapy. Propensity score matching balanced these characteristics and generated a cohort comprising 56 patients from each group. In the matched cohort, the 5 year bRFS of the WPRT group was significantly higher than that of the PBRT group (65.9 vs. 42.2%, *p* = 0.017). Multivariate analysis revealed that WPRT was an independent prognostic factor for bRFS (hazard ratio: 0.45, 95% confidence interval: 0.26–0.75, *p* = 0.002). This benefit of WPRT on bRFS was maintained in subgroup analyses, especially in patients with preoperative PSA level ≤20 ng/mL or pre-SRT PSA level ≥0.4 ng/mL.

**Conclusions:**

These data suggest that, following radical prostatectomy, elective WPRT during SRT may improve bRFS compared with PBRT in selected patients. Patients with preoperative PSA level ≤20 ng/mL or pre-SRT PSA level ≥0.4 ng/mL represent a potential subgroup who benefit most from receiving WPRT. Results of prospective randomized trials are awaited to confirm this finding.

## Introduction

Salvage radiotherapy (SRT) of the prostate bed in patients with biochemical recurrence (BCR) after radical prostatectomy (RP) for the treatment of prostate cancer is associated with higher rates of biochemical control as well as lower rates of distant metastases, cancer-specific mortality, and all-cause mortality in some patients [1–3]. Consequently, its use has been included in guidelines developed by the American Urological Association in collaboration with the American Society for Radiation Oncology [4]. However, more than half of all patients treated with SRT will experience disease progression [1, 5, 6] and more intensive treatment approaches are required to improve outcomes in these patients.

Extending the radiation field to the whole pelvis (whole pelvic radiotherapy, WPRT) in addition to androgen-deprivation therapy (ADT) improved progression-free survival among high-risk patients with an intact prostate [7]. This strategy could also be a rational approach to improving outcomes among patients with BCR after RP and could also improve outcomes in patients with a high risk of pelvic nodal metastasis. A randomized study (RTOG 0534 SPPORT trial) is currently ongoing to investigate the role of WPRT and ADT during SRT, and several retrospective studies have been published, although the conclusions are conflicting [8–11]. These retrospective studies were conducted over a long period of time, beginning in the 1980s, and include inconsistencies in terms of Gleason grading, as well as diagnostic and therapeutic techniques. Therefore, these data may not reflect current practice in patients treated with SRT.

Here, we conducted a retrospective comparison of outcomes in terms of biochemical control as a primary endpoint from two institutions that favor different treatment approaches, offering either prostate bed radiotherapy (PBRT) or WPRT.

## Materials and methods

Data were reviewed from 345 consecutive patients with surgically staged prostate cancer treated with postoperative radiotherapy (RT) after RP between 2005 and 2012 at the Seoul National University Bundang Hospital or Asan Medical Center. None of the patients showed any clinical evidence of distant metastases before they received SRT. Current study was conducted in accordance with the standards and regulations of Korean Good Clinical Practice. Investigators are authorized to proceed the study with approvals of the institutional review board of Seoul National University Bundang Hospital and Asan Medical Center. The requirement for informed consent to participate the study was waived due to its retrospective design.

From this 345 patients in data repository, patients with pathologic lymph node metastases at the time of RP (n = 27), adjuvant RT (n = 18), ADT initiated either prior to RP or >6 months prior to SRT (n = 20), a Roach score ≤15% for the risk of lymph node involvement (n = 3), and clinical follow-up period less than 1 year (n = 4) were excluded. The Roach equation for lymph node involvement, as well as its recommend cutoff value, has been largely adopted in clinical practice and in major randomized trials, such as RTOG 9413 [7, 12]. As we aimed to analyze the impact of elective WPRT compared with PBRT, patients were excluded if they did not receive pelvic lymph node dissection (PLND; n = 36), if they had either clinically suspicious local recurrence (n = 6) or lymph node metastases (n = 12) at the time of SRT, or if they received either WPRT or PBRT discordant with the institution’s preference (n = 28). Ultimately, a total of 191 patients with lymph node-negative prostate cancer patients who were treated with SRT for biochemical relapse after RP were included in this study (data in S1 Dataset).

All patients underwent pre-SRT evaluation with pelvic computed tomography, bone scan and laboratory tests, which included assessment of PSA levels. Multiparametric magnetic resonance imaging was performed in 113 patients (59%). Patients subsequently underwent either PBRT or WPRT. Details of the radiation technique used have been described previously [13–15]. In brief, the clinical target volume (CTV) of WPRT included prostate bed, seminal vesicle, and presacral, obturator, internal iliac, and external iliac nodal regions. The upper border of the CTV was the level of the common iliac bifurcation, which was generally located or immediately above the L5–S1 interspace. The planning target volume was a 3 mm expansion posteriorly and 5–7 mm expansion for the remainders of the CTV. The whole pelvis was irradiated with a total dose of 46 Gy in 23 fractions in most cases. Radiation to the whole pelvis was followed by a boost dose of 20 Gy in 10 fractions in most cases. The median total dose to prostate bed for patients receiving WPRT was 66.0 Gy (range 64.0–70.0 Gy). Meanwhile, for patients treated with PBRT, a total dose of 64.8–70.2 Gy (median 66.0 Gy) was delivered in daily fractional dose of 1.8–2.0 Gy to the prostate bed. After completion of SRT, patients were monitored by digital rectal examination and PSA testing every 3 months for 2 years and at least every 6 months thereafter. Imaging studies were undertaken as clinically indicated. Biochemical relapse post-SRT was defined as two consecutive PSA levels >0.2 ng/mL.

To adjust for significant imbalances in baseline characteristics between the PBRT and WPRT groups, propensity score matching was performed. This approach can be applied to minimize selection bias in observational data [16]. The propensity score of each patient was calculated using multivariate logistic regression for WPRT with the baseline covariates, which included age at SRT, preoperative PSA level, pathologic Gleason scores (pGS), extracapsular extension, seminal vesicle invasion (SVI), surgical margin status, pre-SRT PSA level, total radiation dose to the prostate bed, number of harvested lymph nodes, the use of ADT, and the duration of ADT. The biochemical relapse-free survival (bRFS) was calculated from the beginning of SRT to the date of the second PSA reading of >0.2 ng/mL. Kaplan–Meier analysis was conducted to estimate bRFS with and without propensity score matching. A log-rank test was used to compare differences in bRFS by treatment method. For multivariate analysis for bRFS, the Cox proportional hazards analysis was performed. Variables with *P*-values <0.1 in the univariate analyses and clinically important variables were included as co-variables in the multivariate analysis. Two-sided *P*-values <0.05 were considered to indicate statistical significance. All analyses were carried out with statistical program R (R Foundation for Statistical Computing, Vienna, Austria. http://www.r-project.org/).

## Results

Data from 191 patients were included in the analysis (PBRT, n = 83; WPRT, n = 108). The median follow-up period was 66 months with an interquartile range (IQR) of 53–89 months. The median age at RP and SRT was 66 and 67 years, respectively; the median total prostate bed dose was 66.0 Gy, and median pre-SRT PSA level was 0.550 ng/mL. Intensity modulated radiotherapy and three-dimensional conformal radiotherapy were used in 71% and 29% of patients, respectively. WPRT was offered to 44% of patients with a pGS of 7 and 72% with a pGS of 8–10. ADT was administered concurrently with SRT in 103 (54%) patients (56% pGS 7, 51% pGS 8–10). Median duration of ADT was 17.0 months (IQR 9.7–24.0 months). Median number of nodes dissected was five (IQR 3–7). Details of descriptive statistics relating to patient, tumor, and treatment characteristics for the entire cohort (n = 191), as well as the propensity score-matched cohort (n = 112), are summarized in Table 1. In the entire cohort, there were significant imbalances in these characteristics between the two groups. Briefly, patients who underwent WPRT had higher pGS as well as higher rates of pre-SRT PSA levels >0.5 ng/mL, and lower rates of concurrent ADT. Propensity score matching resulted in a cohort of 56 patients in each group. In the matched cohorts, there were no between-group differences with respect to patient, tumor, and treatment characteristics.

**Table 1:** Baseline variables before and after propensity score matching, stratified by radiation field (PBRT vs. WPRT).

	Entire cohort			Propensity score-matched cohort		
	PBRT	WPRT		PBRT	WPRT	
	(N = 83)	(N = 108)	*P*-value	(N = 56)	(N = 56)	*P*-value
Age at SRT (years)	68 (63–73)	67 (61–71)	0.105	67 (62–73)	67 (63–72)	0.859
Preoperative PSA (ng/mL)						
Median (IQR)	16.8 (10.5–28.6)	16.7 (9.7–32.5)	0.268	16.6 (10.3–28.3)	19.0 (12.5–33.7)	0.657
<10	17 (20.5%)	28 (25.9%)	0.661	12 (21.4%)	9 (16.1%)	0.671
10–20	31 (37.3%)	36 (33.3%)		23 (41.1%)	22 (39.3%)	
>20	35 (42.2%)	44 (40.7%)		21 (37.5%)	25 (44.6%)	
Pathologic Gleason score			0.000			1.000
7	59 (71.1%)	46 (42.6%)		39 (69.6%)	39 (69.6%)	
8–9	24 (28.9%)	62 (57.4%)		17 (30.4%)	17 (30.4%)	
Harvested lymph nodes (n)	5.0 (4.0–8.0)	4.0 (3.0–6.0)	0.164	4.0 (3.3–7.0)	4.0 (3.0–6.8)	0.680
Extracapsular extension			0.391			0.834
No	23 (27.7%)	23 (21.3%)		17 (30.4%)	15 (26.8%)	
Yes	60 (72.3%)	85 (78.7%)		39 (69.6%)	41 (73.2%)	
Seminal vesicle invasion			1.000			0.678
No	52 (62.7%)	67 (62.0%)		38 (67.9%)	41 (73.2%)	
Yes	31 (37.3%)	41 (38.0%)		18 (32.1%)	15 (26.8%)	
Involved surgical margin			0.621			0.692
No	27 (32.5%)	40 (37.0%)		18 (32.1%)	21 (37.5%)	
Yes	56 (67.5%)	68 (63.0%)		38 (67.9%)	35 (62.5%)	
pre-SRT PSA (ng/mL)						
Median (IQR)	0.46 (0.27–0.88)	0.60 (0.38–0.86)	0.263	0.51 (0.30–0.98)	0.61 (0.37–0.91)	0.638
<0.5	46 (55.4%)	39 (36.1%)	0.002	28 (50.0%)	20 (35.7%)	0.135
0.5–1.0	19 (22.9%)	52 (48.1%)		15 (26.8%)	25 (44.6%)	
>1.0	18 (21.7%)	17 (15.7%)		13 (23.2%)	11 (19.6%)	
Total dose to prostate bed (Gy)	66.0 (64.8–66.6)	66.0 (66.0–66.0)	0.240	66.0 (64.8–70.0)	66.0 (66.0–66.0)	0.946
Total dose to whole pelvis (Gy)	–	46.0 (44.0–46.0)	–	–	46.0 (44.0–46.0)	–
Use of ADT			0.010			0.705
No	29 (34.9%)	59 (54.6%)		29 (51.8%)	26 (46.4%)	
Yes	54 (65.1%)	49 (45.4%)		27 (48.2%)	30 (53.6%)	
Duration of ADT (months)	15.9 (9.0–25.1)	17.2 (13.6–21.1)	0.640	15.6 (8.5–25.8)	17.1 (10.9–20.9)	0.699

Categorical variables are presented as n (%), continuous variables are presented as median (interquartile range).

Abbreviations: PBRT = prostate bed only radiotherapy; WPRT = whole pelvic radiotherapy; SRT = salvage radiotherapy; PSA = prostate-specific antigen; IQR = interquartile range; ADT = androgen-deprivation therapy.

**Fig 1:**
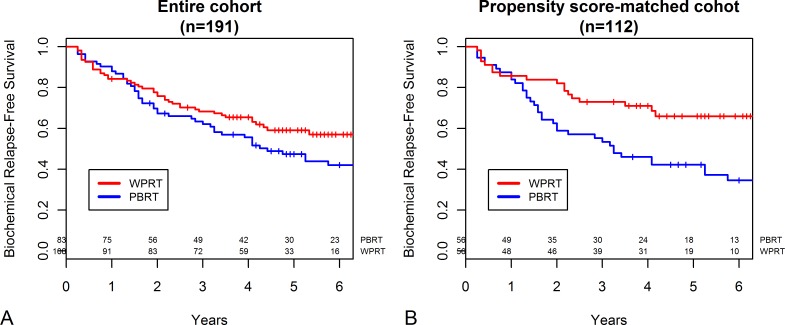
**Biochemical relapse-free survival probability stratified based on receipt of whole pelvic radiotherapy (WPRT) or prostate bed-only radiotherapy (PBRT) in (A) the entire cohort (n = 191) and (B) the propensity score-matched cohort (n = 112)**.

Fig 1 shows the Kaplan–Meier survival curves of the entire and matched cohorts. The 5 year bRFS rate of the WPRT vs. PBRT groups in the initial unmatched cohort were 59.1% vs. 47.4% (*P* = 0.247, Fig 1A). In the matched cohort, the 5 year bRFS rate of the WPRT group was significantly higher than that of the PBRT group (65.9% vs. 42.2%, *P* = 0.017, Fig 1B).

Table 2 summarizes the results of univariate and multivariate analysis for bRFS. In the univariate analysis, involved surgical margin, no SVI, lower pre-SRT PSA, and the addition of ADT to SRT were significantly associated with improved bRFS. After multivariate analysis, age at SRT (adjusted hazard ratio [HR] 0.97, 95% confidence interval [CI] 0.93–0.99, *P* = 0.040), pre-SRT PSA level (adjusted HR 4.25, 95% CI 2.35–7.70, *P* < 0.001), use of WPRT (adjusted HR 0.45, 95% CI 0.26–0.75, *P* = 0.002), and the addition of ADT to SRT (adjusted HR 0.31, 95% CI 0.18–0.52, *P* < 0.001) retained a significant association with bRFS.

**Table 2:** Univariate and multivariate analysis for biochemical relapse-free survival.

	Univariate			Multivariate		
	HR	(95% CI)	*P*-value	HR	(95% CI)	*P*-value
Age at SRT (continuous)	0.99	(0.96–1.02)	0.435	0.97	(0.93–0.99)	0.040
Preoperative PSA (ng/mL)						
≤20		RL	–		RL	–
>20	0.96	(0.63–1.45)	0.830	1.03	(0.66–1.60)	0.894
Pathologic Gleason score						
7		RL	–		RL	–
8	1.03	(0.56–1.91)	0.927	1.39	(0.69–2.83)	0.358
9	1.57	(1.00–2.46)	0.051	1.49	(0.91–2.46)	0.117
Harvested lymph nodes (n)						
>4		RL	–		–	–
≤4	1.15	(0.77–1.74)	0.496			
Extracapsular extension						
No		RL	–		RL	–
Yes	1.10	(0.68–1.78)	0.700	1.26	(0.68–2.32)	0.469
Seminal vesicle invasion						
No		RL	–		RL	–
Yes	1.55	(1.03–2.35)	0.037	1.43	(0.87–2.33)	0.156
Involved surgical margin						
No		RL	–		RL	–
Yes	0.57	(0.37–0.86)	0.007	0.65	(0.39–1.08)	0.095
pre-SRT PSA (ng/mL)						
<0.5		RL	–		RL	–
0.5–1.0	1.21	(0.75–1.96)	0.439	1.73	(1.01–2.96)	0.047
>1.0	2.56	(1.53–4.29)	<0.001	4.25	(2.35–7.70)	<0.001
Total dose to prostate bed (Gy)						
<70		RL	–		RL	–
≥70	1.00	(0.61–1.64)	0.987	0.57	(0.31–1.04)	0.064
Radiation field						
PBRT		RL	–		RL	–
WPRT	0.79	(0.52–1.19)	0.252	0.45	(0.26–0.75)	0.002
Use of ADT						
No		RL	–		RL	–
Yes	0.49	(0.33–0.75)	0.001	0.31	(0.18–0.52)	<0.001

Abbreviations: HR = hazard ratio; CI = confidence interval; SRT = salvage radiotherapy; PSA = prostate-specific antigen; RL = referent level; PBRT = prostate bed only radiotherapy; WPRT = whole pelvic radiotherapy; ADT = androgen-deprivation therapy.

**Fig 2:**
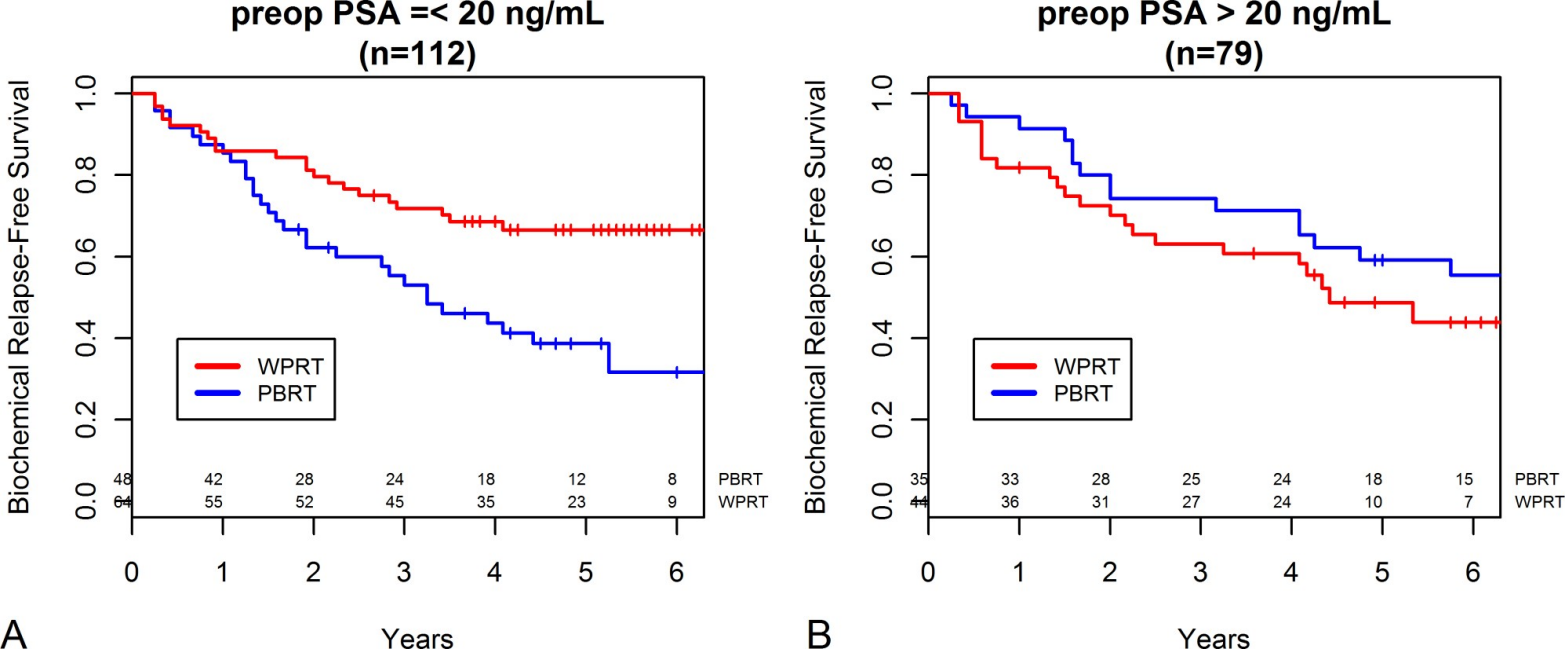
**Biochemical relapse-free survival probability stratified based on receipt of whole pelvic radiotherapy (WPRT) or prostate bed-only radiotherapy (PBRT) in (A) patients with preoperative PSA level ≤20 ng/mL (n = 112) and (B) patients with preoperative PSA level >20 ng/mL (n = 79)**.

Subgroup analyses were performed to identify the population that experienced the greatest benefit from WPRT. When patients were divided by preoperative PSA level, 112 patients had preoperative PSA level ≤20 ng/mL and 79 patients had >20 ng/mL. For patients with preoperative PSA level ≤20 ng/mL, those who received WPRT had a significantly higher 5 year bRFS rate than those who did not (66.5% vs. 38.7%, *P* = 0.007, Fig 2A). Meanwhile, WPRT conferred no benefit over PBRT in patients with preoperative PSA level >20 ng/mL. The 5 year bRFS rate of the WPRT vs. PBRT were 48.7% vs. 59.1% (*P* = 0.214, Fig 2B).

**Fig 3:**
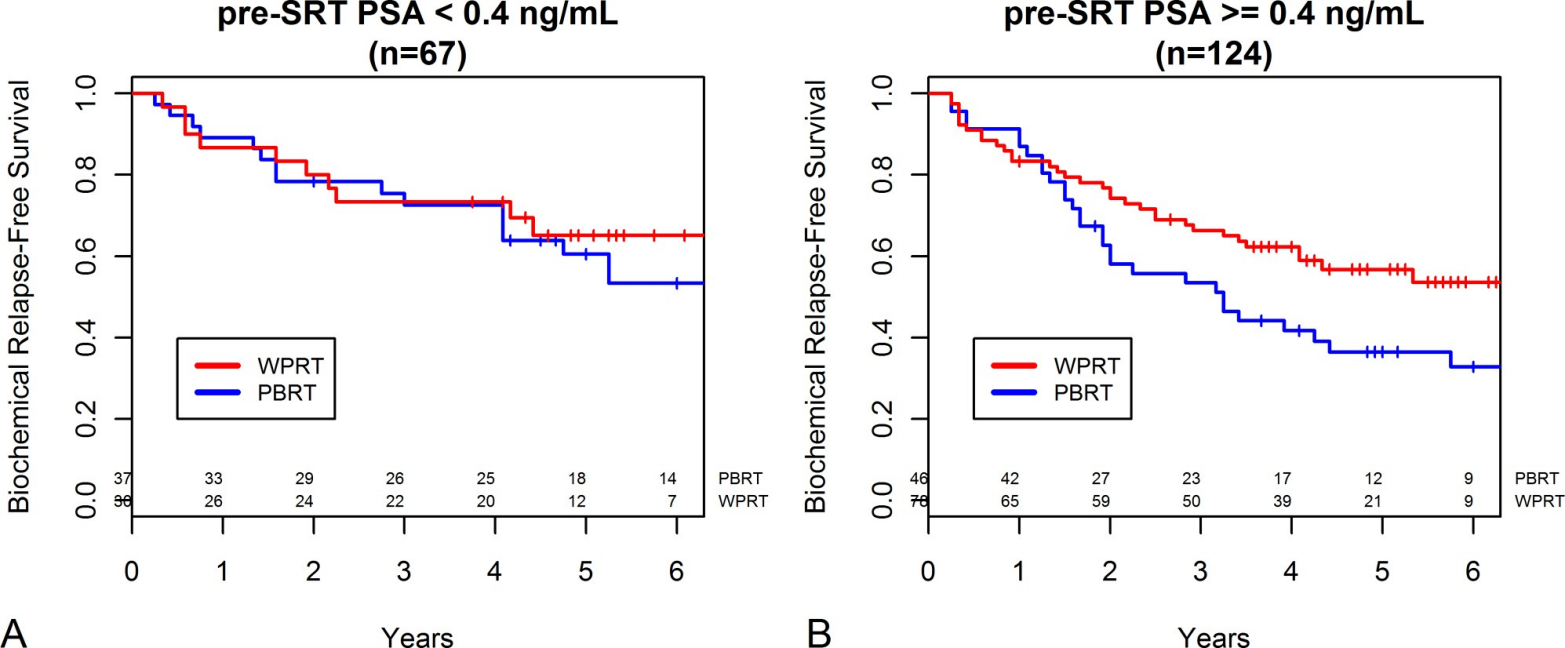
**Biochemical relapse-free survival probability stratified based on receipt of whole pelvic radiotherapy (WPRT) or prostate bed-only radiotherapy (PBRT) in (A) patients with pre-salvage radiotherapy PSA level <0.4 ng/mL (n = 67) and (B) patients with pre-salvage radiotherapy PSA level ≥0.4 ng/mL (n = 124)**.

When patients were grouped by pre-SRT PSA level of 0.4 ng/mL based on the previous observational study [8] which reported pre-SRT PSA level ≥0.4 ng/mL to be associated with improved bRFS with WPRT, 67 patients had pre-SRT PSA level <0.4 ng/mL and 124 patients had ≥0.4 ng/mL. WPRT conferred no benefit over PBRT in patients with pre-SRT PSA level <0.4 ng/mL (the 5 year bRFS 65.1% vs. 60.5%, *P* = 0.811, Fig 3A). For patients with pre-SRT PSA level ≥0.4 ng/mL, there was a significant benefit of WPRT compared to PBRT (the 5 year bRFS 56.8% vs. 36.5%,; *P* = 0.043, Fig 3B).

Meanwhile, WPRT failed to show significant benefit in patients with SVI (*P* = 0.348) or involved surgical margin (*P* = 0.083).

## Discussion

Approximately 30% of patients treated with RP for prostate cancer experience disease recurrence, often first evidenced by rising PSA levels [17, 18]. SRT reduces the incidence of distant metastases, cancer-specific mortality, and all-cause mortality compared with observation only [1–3]. However, the optimal radiation target volume, which is based on individualized risk, has not been established in this setting, and RT to the prostatic bed only is recommended by several consensus guidelines [19, 20]. Limitations to establishing the anatomic location of the occult disease with current imaging modalities also hinder the selection of an appropriate target volume. A recent study reports patterns of recurrence after PBRT, as identified by C-11 choline positron emission tomography [21]. In this study, the majority of recurrences were located outside the prostate fossa and in the pelvic nodal area, particularly inferior to the aortic bifurcation. Although these findings should be interpreted with caution, being hypothesis-generating at best, they did suggest a possible need for nodal irradiation. For selected, high-risk patients, WPRT is often used to irradiate pelvic lymph nodes, which may harbor occult metastases [11, 22]. This study furthers understanding of the clinical implications of BCR after RP and the treatment effect of WPRT during SRT, confirming the results of previous studies demonstrating that WPRT (with or without ADT) can reduce the incidence of subsequent secondary biochemical relapse [11, 13]. With regard to the potential increase in toxicity following WPRT compared with PBRT in the postprostatectomy RT setting, several studies have demonstrated that the risk of developing late GI toxicity is significantly reduced with intensity modulated radiotherapy compared with three-dimensional conformal radiotherapy [15, 23, 24].

In the present study, WPRT was significantly associated with improved 5 year bRFS compared with PBRT after propensity score matching (65.9% vs. 42.2%, *P* = 0.017). In multivariate analysis, when adjusting for other clinically meaningful risk factors (such as age, pGS, ECE, SVI, surgical margin status, use of ADT, RT dose, and preoperative and pre-SRT PSA level) WPRT was seen to be independently associated with a 55% reduction in the risk of biochemical relapse. Similarly, concurrent ADT use was also associated with reduced biochemical relapse in the multivariate analysis (adjusted HR 0.31, 95% CI 0.18–0.52, *P* < 0.001). Regarding the synergistic effect of RT and ADT, it is well known that ADT inhibits non-homologous end joining, an important DNA repair process [25]. A recent study also demonstrated that RT results in androgen receptor (AR) upregulation in various *in vitro* and *in vivo* prostate cancer models, providing further evidence of synergism between ADT and RT [26]. The study also assessed AR-regulated hK2 protein, demonstrating AR upregulation in approximately 20% of patients receiving definitive RT. The efficacy of this approach, using concurrent ADT during SRT, has also been demonstrated in two recent randomized trials, RTOG 9601 [27] and GETUG-AFU 16 [28]. RTOG 9601 compared the outcomes of patients receiving long-term ADT with those of patients receiving placebo during SRT and showed an improvement in overall survival and distant metastases with long-term ADT [27]. The GETUG-AFU 16 study demonstrated that short-term ADT administered with SRT improved progression-free survival [28]. However, the majority of patients in these trials were treated by PBRT, rather than WPRT. Therefore, whether ADT is also beneficial with WPRT remains uncertain. This question will be answered by the RTOG 0534 SPPORT trial, which is an ongoing study of patients randomly assigned into three treatment groups: PBRT alone, PBRT plus short-term ADT, and WPRT plus short-term ADT. The first report of an interim analysis of RTOG 0534 SPPORT trial was presented recently at the 60th Annual Meeting of the American Society for Radiation Oncology [29]. At 5 years following treatment, freedom from progression rates in the interim analysis group were 71% for PBRT alone, 83% for PBRT plus ADT, and 89% for WPRT plus ADT (*P* < 0.0001). Freedom from progression was defined as a PSA nadir + 2.0 ng/mL, clinical failure, or death from any cause.

The present study has several limitations. First, the retrospective design means that it is subject to inherent bias. Adjustment for possible confounding factors was made by propensity score matching, although it is possible that unknown confounding factors may persist. Second, the median follow-up time of 66 months is relatively short for patients with prostate cancer, which has a long natural history; during this time, the effects of ADT could be over-riding the biochemical control resulting from WPRT. Third, the cutoff for PLND and the extent of PLND were not standardized among different surgeons in the current study. In most cases, limited PLND was performed. Therefore, a certain proportion of patients who received sub-optimal PLND might be included in the current study. The median number of pelvic lymph nodes removed was five, indicating possible understaging. Although pelvic lymph node dissection is the most accurate method of determining nodal staging, its therapeutic benefit remains debatable and no consistent conclusion has been reached [30–32]. Finally, toxicity data were not reliable due to the retrospective nature of the study and therefore precluded any meaningful analysis.

## Conclusions

Elective WPRT during SRT may improve bRFS compared with PBRT in selected patients. Patients with preoperative PSA level ≤20 ng/mL or pre-SRT PSA level ≥0.4 ng/mL represent a potential subgroup who benefit most from receiving WPRT. Results of prospective randomized trials are awaited to confirm the benefit of elective WPRT during SRT.

## Supporting information

S1 Dataset Original data for analysis. (XLSX)

## References

[1] Trock BJ, Han M, Freedland SJ, Humphreys EB, DeWeese TL, Partin AW, et al Prostate cancer-specific survival following salvage radiotherapy vs observation in men with biochemical recurrence after radical prostatectomy. JAMA. 2008; 299: 2760–2769. 10.1001/jama.299.23.2760 18560003 PMC3076799

[2] Cotter SE, Chen MH, Moul JW, Lee WR, Koontz BF, Anscher MS, et al Salvage radiation in men after prostate-specific antigen failure and the risk of death. Cancer. 2011; 117: 3925–3932. 10.1002/cncr.25993 21437885

[3] Boorjian SA, Karnes RJ, Crispen PL, Rangel LJ, Bergstralh EJ, Blute ML. Radiation therapy after radical prostatectomy: Impact on metastasis and survival. J Urol. 2009; 182: 2708–2714. 10.1016/j.juro.2009.08.027 19836762

[4] Thompson IM, Valicenti RK, Albertsen P, Davis BJ, Goldenberg SL, Hahn C, et al Adjuvant and salvage radiotherapy after prostatectomy: AUA/ASTRO guideline. J Urol. 2013; 190: 441–449. 10.1016/j.juro.2013.05.032 23707439

[5] Stephenson AJ, Scardino PT, Kattan MW, Pisansky TM, Slawin KM, Klein EA, et al Predicting the outcome of salvage radiation therapy for recurrent prostate cancer after radical prostatectomy. J Clin Oncol. 2007; 25: 2035–2041. 10.1200/JCO.2006.08.9607 17513807 PMC2670394

[6] Choo R. Salvage radiotherapy for patients with PSA relapse following radical prostatectomy: issues and challenges. Cancer Res Treat. 2010; 42: 1–11. 10.4143/crt.2010.42.1.1 20369045 PMC2848745

[7] Roach M 3rd, DeSilvio M, Lawton C, Uhl V, Machtay M, Seider MJ, et al Phase III trial comparing whole-pelvic versus prostate-only radiotherapy and neoadjuvant versus adjuvant combined androgen suppression: Radiation Therapy Oncology Group 9413. J Clin Oncol. 2003; 21: 1904–1911. 10.1200/JCO.2003.05.004 12743142

[8] Moghanaki D, Koontz BF, Karlin JD, Wan W, Mukhopadhay N, Hagan MP, et al Elective irradiation of pelvic lymph nodes during postprostatectomy salvage radiotherapy. Cancer. 2013; 119: 52–60. 10.1002/cncr.27712 22736478

[9] Kim BS, Lashkari A, Vongtama R, Lee SP, Parker RG. Effect of pelvic lymph node irradiation in salvage therapy for patients with prostate cancer with a biochemical relapse following radical prostatectomy. Clin Prostate Cancer. 2004; 3: 93–97. 10.3816/CGC.2004.n.018 15479492

[10] Spiotto MT, Hancock SL, King CR. Radiotherapy after prostatectomy: Improved biochemical relapse-free survival with whole pelvic compared with prostate bed only for high-risk patients. Int J Radiat Oncol Biol Phys. 2007; 69: 54–61. 10.1016/j.ijrobp.2007.02.035 17459606

[11] Ramey SJ, Agrawal S, Abramowitz MC, Moghanaki D, Pisansky TM, Efstathiou JA, et al Multi-institutional evaluation of elective nodal irradiation and/or androgen deprivation therapy with postprostatectomy salvage radiotherapy for prostate cancer. Eur Urol. 2017 10.1016/j.eururo.2017.10.009 29128208

[12] Lawton CA, DeSilvio M, Roach M 3rd, Uhl V, Kirsch R, Seider M, et al An update of the phase III trial comparing whole pelvic to prostate only radiotherapy and neoadjuvant to adjuvant total androgen suppression: Updated analysis of RTOG 94–13, with emphasis on unexpected hormone/radiation interactions. Int J Radiat Oncol Biol Phys. 2007; 69: 646–655. 10.1016/j.ijrobp.2007.04.003 17531401 PMC2917177

[13] Song C, Kang HC, Kim JS, Eom KY, Kim IA, Chung JB, et al Elective pelvic versus prostate bed-only salvage radiotherapy following radical prostatectomy: A propensity score-matched analysis. Strahlenther Onkol. 2015; 191: 801–809. 10.1007/s00066-015-0872-9 26159555

[14] Joo JH, Kim YJ, Kim YS, Cho YP, Lee HY, Jeong CY, et al Analysis of prostate bed motion using an endorectal balloon and cone beam computed tomography during postprostatectomy radiotherapy. Onco Targets Ther. 2016; 9: 3095–3100. 10.2147/OTT.S98112 27307750 PMC4888733

[15] Byun SJ, Kim YS, Ahn H, Kim CS. Image-guided, whole-pelvic, intensity-modulated radiotherapy for biochemical recurrence following radical prostatectomy in high-risk prostate cancer patients. PLoS One. 2018; 13: e0190479 10.1371/journal.pone.0190479 29320570 PMC5761863

[16] D'Agostino RB Jr., D'Agostino RB Sr. Estimating treatment effects using observational data. JAMA. 2007; 297: 314–316. 10.1001/jama.297.3.314 17227985

[17] Toussi A, Stewart-Merrill SB, Boorjian SA, Psutka SP, Thompson RH, Frank I, et al Standardizing the definition of biochemical recurrence after radical prostatectomy-what prostate specific antigen cut point best predicts a durable increase and subsequent systemic progression? J Urol. 2016; 195: 1754–1759. 10.1016/j.juro.2015.12.075 26721226

[18] Koo KC, Cho JS, Bang WJ, Lee SH, Cho SY, Kim SI, et al Cancer-specific mortality among Korean men with localized or locally advanced prostate cancer treated with radical prostatectomy versus radiotherapy: A multi-center study using propensity scoring and competing risk regression analyses. Cancer Res Treat. 2018; 50: 129–137. 10.4143/crt.2017.004 28279064 PMC5784628

[19] Michalski JM, Lawton C, El Naqa I, Ritter M, O'Meara E, Seider MJ, et al Development of RTOG consensus guidelines for the definition of the clinical target volume for postoperative conformal radiation therapy for prostate cancer. Int J Radiat Oncol Biol Phys. 2010; 76: 361–368. 10.1016/j.ijrobp.2009.02.006 19394158 PMC2847420

[20] Poortmans P, Bossi A, Vandeputte K, Bosset M, Miralbell R, Maingon P, et al Guidelines for target volume definition in post-operative radiotherapy for prostate cancer, on behalf of the EORTC Radiation Oncology Group. Radiother Oncol. 2007; 84: 121–127. 10.1016/j.radonc.2007.07.017 17706307

[21] Parker WP, Evans JD, Stish BJ, Park SS, Olivier K, Choo R, et al Patterns of recurrence after postprostatectomy fossa radiation therapy identified by C-11 choline positron emission tomography/computed tomography. Int J Radiat Oncol Biol Phys. 2017; 97: 526–535. 10.1016/j.ijrobp.2016.11.014 28126302 PMC5308881

[22] Chang AR, Park W. Radiotherapy in prostate cancer treatment: Results of the patterns of care study in Korea. Radiat Oncol J. 2017; 35: 25–31. 10.3857/roj.2016.01984 28292006 PMC5398354

[23] Alongi F, Fiorino C, Cozzarini C, Broggi S, Perna L, Cattaneo GM, et al IMRT significantly reduces acute toxicity of whole-pelvis irradiation in patients treated with post-operative adjuvant or salvage radiotherapy after radical prostatectomy. Radiother Oncol. 2009; 93: 207–212. 10.1016/j.radonc.2009.08.042 19766338

[24] Goenka A, Magsanoc JM, Pei X, Schechter M, Kollmeier M, Cox B, et al Improved toxicity profile following high-dose postprostatectomy salvage radiation therapy with intensity-modulated radiation therapy. Eur Urol. 2011; 60: 1142–1148. 10.1016/j.eururo.2011.08.006 21855208

[25] Polkinghorn WR, Parker JS, Lee MX, Kass EM, Spratt DE, Iaquinta PJ, et al Androgen receptor signaling regulates DNA repair in prostate cancers. Cancer Discov. 2013; 3: 1245–1253. 10.1158/2159-8290.CD-13-0172 24027196 PMC3888815

[26] Spratt DE, Evans MJ, Davis BJ, Doran MG, Lee MX, Shah N, et al Androgen receptor upregulation mediates radioresistance after ionizing radiation. Cancer Res. 2015; 75: 4688–4696. 10.1158/0008-5472.CAN-15-0892 26432404 PMC4651750

[27] Shipley WU, Seiferheld W, Lukka HR, Major PP, Heney NM, Grignon DJ, et al Radiation with or without antiandrogen therapy in recurrent prostate cancer. N Engl J Med. 2017; 376: 417–428. 10.1056/NEJMoa1607529 28146658 PMC5444881

[28] Carrie C, Hasbini A, de Laroche G, Richaud P, Guerif S, Latorzeff I, et al Salvage radiotherapy with or without short-term hormone therapy for rising prostate-specific antigen concentration after radical prostatectomy (GETUG-AFU 16): A randomised, multicentre, open-label phase 3 trial. Lancet Oncol. 2016; 17: 747–756. 10.1016/S1470-2045(16)00111-X 27160475

[29] The ASCO Post et al. 2018 ASTRO: SPPORT Trial: ADT With or Without Pelvic Lymph Node Radiation in Prostate Cancer. The ASCO Post 30 10 2018 Available from: http://www.ascopost.com/News/59414 Cited 15 March 2019.

[30] Preisser F, Bandini M, Marchioni M, Nazzani S, Tian Z, Pompe RS, et al Extent of lymph node dissection improves survival in prostate cancer patients treated with radical prostatectomy without lymph node invasion. Prostate. 2018; 78: 469–475. 10.1002/pros.23491 29460290

[31] Leyh-Bannurah SR, Budaus L, Zaffuto E, Pompe RS, Bandini M, Briganti A, et al Adherence to pelvic lymph node dissection recommendations according to the National Comprehensive Cancer Network pelvic lymph node dissection guideline and the D'Amico lymph node invasion risk stratification. Urol Oncol. 2018; 36: 81 e17–81 e24. 10.1016/j.urolonc.2017.10.022 29248430

[32] Murphy AM, Berkman DS, Desai M, Benson MC, McKiernan JM, Badani KK. The number of negative pelvic lymph nodes removed does not affect the risk of biochemical failure after radical prostatectomy. BJU Int. 2010; 105: 176–179. 10.1111/j.1464-410X.2009.08707.x 19549117 PMC5508720

